# Perceptual versus motor spatiotemporal interactions in duration reproduction across two hands

**DOI:** 10.1038/srep23365

**Published:** 2016-04-01

**Authors:** Tsuyoshi Kuroda, Makoto Miyazaki

**Affiliations:** 1 Faculty of Informatics, Shizuoka University, 3-5-1 Johoku, Naka-ku, Hamamatsu, Shizuoka, 432-8011, Japan

## Abstract

The possibility of spatiotemporal interactions in motor action that are comparable with the perceptual kappa effect was tested in the present study. In the kappa effect, the empty duration between two successive stimuli is overestimated when the spatial distance between these stimuli is increased. Indeed, when participants reproduced the standard (empty) duration, delivering two tactile stimuli to different hands resulted in a longer reproduced duration than delivering both stimuli to the same hand, regardless of how long the standard was. However, when a spatial factor during motor action (reproduction) was manipulated by letting participants use an identical hand or different hands for two button pushes reproducing the standard, the different-hand condition yielded a shorter reproduced duration than the identical-hand condition when the standard was 1000 ms or more. More specifically, this decrement in the reproduced duration grew linearly with the standard, suggesting that a given space increases the “rate” of an internal timer during motor action. Because each tick of the timer was accelerated, the total error causing an earlier push of the second button was increased with the standard. A pacemaker-counter model was adopted to explain the differences between the perceptual and the motor spatiotemporal interactions.

Interactions between space and time in perception have recently attracted attention from neuroscience researchers^1^,^2^,^3^. *A theory of magnitude*^1^,^4^ is one of the influential theories involved with spatiotemporal processing and indicates that certain physical variables, including duration and spatial distance, are quantified as the same type of mental magnitudes, thus interacting with each other. Indeed, the use of spatial representations helps to address time which is an abstract concept^3^; for example, a horizontal axis is often used to represent time when a time course of events is explained. Moreover, space and time must be combined in certain functions of the brain to enable motion perception as well as motor action, such as reaching and catching a ball. The theory^1^,^4^ posits that the parietal cortex involves a common processing of space, time and other quantities.

Spatiotemporal interactions in perception were already a scientific concern early in the 20th century in the field of psychophysics, where a famous illusion, called the *kappa effect*, was found^5^,^6^,^7^,^8^,^9^,^10^. Price-Williams^8^ conducted a series of experiments in which participants reproduced the duration of an empty time interval marked by two successive flashes. The reproduced duration was longer when the two flashes were located farther away from each other in space. In other words, the duration was overestimated when the spatial distance was increased.

This kappa effect also occurs in the tactile modality^11^,^12^,^13^. Grondin *et al.*^13^ reported that the empty duration was perceived as longer when two tactile stimuli were delivered to different hands than when both stimuli were delivered to the same hand of each participant. In this previous experiment, participants compared the standard (empty) interval of 500 ms with the comparison interval of variable duration. Each of the two stimuli that marked the comparison was delivered to either the left or the right hand. The hand that each stimulus would be delivered to was varied across trials. Thus, the kappa effect is typically produced by manipulating the spatial location of stimuli that participants passively receive. However, in daily activity, people move different parts of the body actively at certain temporal intervals. For example, two arms are moved alternately at a constant time interval for rolling a drum, and walking requires controlling the timing of the alternate movements of two legs. It is possible that time keeping in motor action is modulated by the space between the bodily parts to be moved. Indeed, several neurophysiological studies have indicated that brain regions involving motor controls, such as the cerebellum and the motor cortex, are also activated during sub-second time judgements^14^,^15^. Moreover, the parietal cortex is posited as a source of spatiotemporal interactions^1^,^2^,^4^ and is also posited to involve motor controlls^16^,^17^,^18^. If the neural basis of spatiotemporal interactions as found in the perceptual kappa effect is linked with the functions of these brain regions, it is plausible that such interactions also occur in motor action. This possibility was tested in the present study. More specifically, we examined whether duration reproduction would be modulated when participants used different hands, compared with when using the same hand, for two button pushes reproducing an empty time interval. Notably, in the study of Grondin *et al.*^13^, the hand that each stimulus would be delivered to was manipulated, while in the present investigation, the hand that participants voluntarily moved for reproducing the empty duration was manipulated. In other words, the previous study examined the possibility of spatiotemporal interactions in perception, whereas the present study examined the possibility of spatiotemporal interactions in motor action.

Experiment 1 consisted of two sessions. A schematic explanation of each session is shown in Fig. 1. The “perceptual” session was conducted to replicate the kappa effect in perception. The standard duration of 500, 1000, or 1500 ms was marked by the onsets of two successive stimuli, and the location of each stimulus was manipulated; the two stimuli were delivered to the same hand or to different hands of each participant. Each stimulus, lasting 1 ms, was given by a cuboid solenoid vibrator that was attached to the back of each hand and laid along the second metacarpal bone; thus, participants perceived each stimulus around this bone on the back of the hand (Fig. 2). After the presentation of the two stimuli, the participants reproduced the standard duration by pushing a button twice with the index finger of one hand. The first push and the second push delimited the onset and the offset of duration, respectively. Note that we adopted the above stimulus location because it was close to the index finger but did not disturb the movement of the index finger. If the typical kappa effect takes place, delivering two stimuli to different hands should cause an overestimation of the standard duration, expressed by the elongation of the reproduced duration, compared with delivering both stimuli to an identical hand.

The “motor” session was of interest for the current arguments. The hand for reproduction was manipulated in this session; an identical hand or different hands were used for two button pushes. The location of each button is shown in Fig. 2. There are two hypotheses here: (1) Perceptual spatiotemporal interactions that result in the kappa effect are sourced from the brain areas such as the motor cortex and the parietal cortex involving motor action as mentioned above, and thus the similar spatiotemporal interactions are found in motor action; (2) The perceptual kappa effect is not linked to these brain areas, and thus no specific type of spatiotemporal interactions is found in motor action.

If the first hypothesis is adopted, the two button pushes with different hands should cause an overestimation of the ongoing duration during reproduction, compared with those with the same hand. This hypothesis then predicts a *shorter* reproduced duration in the different-hand than the identical-hand condition. Note that it does not predict a longer reproduced duration; because the ongoing duration is overestimated during reproduction, participants finish the reproduction (push the second button) earlier, resulting in a shorter reproduced duration. Alternatively, if the second hypothesis is adopted, the reproduced duration should be unchanged regardless of whether the same hand or different hands are used for the two button pushes.

There was a potential problem in the technical procedures of Experiment 1. Participants were informed of the hand that was to be used for reproduction at the beginning of each trial in the motor session. The same procedure was adopted in the perceptual session, although the same hand was used throughout a block of trials, to maintain the consistency of the task (and thereby avoid confusing the participants). In other words, the participants were not informed of which hand each stimulus would be delivered to in the perceptual session. They consequently could not expect, or pay attention to, the tactile location (hand) that would be stimulated. However, in the motor session, they were informed of the hand to be moved and thus could pay attention to that location before reproduction. Therefore, the perceptual and motor sessions might have differed in the expectancy as well as the attentional focus on the hand that was stimulated/moved. Indeed, both expectancy and attentional focus are known to be factors influencing duration processing^19^,^20^. Moreover, Grondin^21^ suggested that the spatial effects on perceived duration in the visual modality disappeared when participants could expect the location of the first stimulus that marked the beginning of the empty duration. If there are some differences between the results of the perceptual session and those of the motor session, these differences might be attributed to the expectancy as well as the attentional focus, rather than to the property of spatiotemporal interactions in these modalities. We thus conducted Experiment 2 with only the perceptual session in which the participants were pre-informed of (and thus could expect) the hand that would be stimulated. If the perceptual- vs. motor-session differences in Experiment 1 are attributed to the expectancy or the attentional focus, the results of Experiment 2 should be similar to those of the motor session in Experiment 1 (where participants could pay attention to the hand to be moved), rather than to those of the perceptual session in Experiment 1 (where participants could not pay attention to the hand that would be stimulated). Alternatively, if neither the expectancy nor the attentional focus affects time keeping in the current experiments, the perceptual sessions of both Experiments 1 and 2 should show almost the same results.

In brief, the possibility of spatiotemporal interactions in motor action, comparable with the perceptual kappa effect, was tested in Experiment 1, and the possibility of a technical problem in Experiment 1 was tested in Experiment 2.

## Results

### Experiment 1

The perceptual session was conducted to replicate the occurrence of the kappa effect in perception. The experimental conditions were based on a two-way factorial design with repeated measures. The first factor consisted of the three standard durations (500, 1000 and 1500 ms) and the second consisted of the four locations (LL, LR, RL and RR). For convenience, we call the LR and RL conditions the “with-space” conditions because a space was given between two stimuli; moreover, we call the LL and RR conditions the “without-space” conditions because no space was given between two stimuli. The mean reproduced duration for each condition is shown in Fig. 3. In general, the with-space (LR and RL) conditions resulted in a longer reproduction than the without-space (LL and RR) conditions for all standard durations (500, 1000 and 1500 ms). Indeed, a two-way repeated-measures analysis of variance (ANOVA) showed that the location effect, *F*(1.23, 18.39) = 13.076, *p* = 0.001, η_*p*_^2^ = 0.466, as well as the standard effect, *F*(1.38, 20.63) = 563.102, *p* < 0.001, η_*p*_^2^ = 0.974, was significant, whereas the interaction was not significant, *F*(2.47, 37.06) = 0.709, *p* = 0.527, η_*p*_^2^ = 0.045. The post hoc contrasts according to the Scheffé method for the location effect rejected the null hypothesis positing 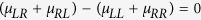, indicating that delivering the two stimuli to different hands caused a significantly longer reproduction than delivering them to an identical hand. Neither 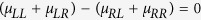 nor 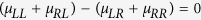 was rejected, indicating that reproduction was not influenced by whether the first stimulus was delivered to the left or the right hand, or by whether the second stimulus was delivered to the left or the right hand.

The motor session was conducted to examine whether spatiotemporal interactions would occur in motor action. As in the perceptual session, the experimental conditions were based on a 3 (standard duration) × 4 (location) design with repeated measures. In the with-space (LR and RL) conditions, different hands were used for two button pushes reproducing the standard duration, and in the without-space (LL and RR) conditions, the same hand was used for the two button pushes. The mean reproduced duration for each condition is shown in Fig. 3. In general, the with-space conditions resulted in a briefer reproduction than the without-space conditions when the standard was 1000 ms or more, although the differences among the mean values seemed small. A two-way repeated-measures ANOVA showed that, whereas the location effect was not significant, *F*(1.86, 27.9) = 3.263, *p* = 0.057, η_*p*_^2^ = 0.179, the interaction, *F*(3.51, 52.59) = 3.940, *p* = 0.010, η_*p*_^2^ = 0.208, as well as the standard effect, *F*(1.28, 19.24) = 591.538, *p* < 0.001, η_*p*_^2^ = 0.975, was significant. When the standard was 1000 ms and 1500 ms, the Scheffé contrasts rejected the null hypothesis positing 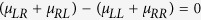, indicating that the two button pushes with different hands caused a significantly briefer reproduction than those with an identical hand. Neither 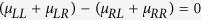 nor 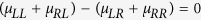 was rejected for all standards, indicating no effects of the first pushed button or of the second pushed button.

No spatial effects were found when the standard was 500 ms, whereas the reproduced duration was shortened in the with-space conditions when the standard was 1000 and 1500 ms for the motor session. Thus, the reproduction error resulting from a given space (LR and RL) relative to no space (LL and RR) might be increased as a function of the standard duration. Indeed, as presented in Fig. 4, the mean spatial-effect amount, estimated with the equation 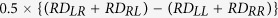, was magnified in a negative direction as the standard duration was lengthened for the motor session (*RD*, reproduced duration). A one-way repeated-measures ANOVA demonstrated no significant effect for the perceptual session, *F*(1.36, 20.36) = 0.803, *p* = 0.417, η_*p*_^2^ = 0.051, whereas it showed a significant effect for the motor session, *F*(2, 30) = 7.472, *p* = 0.002, η_*p*_^2^ = 0.333. For the latter case, the trend analysis^22^ revealed that the spatial-effect amount was linearly changed as a function of the standard duration, *F*_*lin*_(1, 30) = 13.474, *p* < 0.001, but not in a quadratic manner, *F*_*quad*_(1, 30) = 1.470, *p* = 0.235.

### Experiment 2

As explained in the Introduction, the perceptual and motor sessions of Experiment 1 differed in the participant’s expectation as well as the resultant attentional focus^19^,^20^,^21^ on the hand that would be stimulated/moved, which might have led to the differences between the results of these sessions. We thus conducted Experiment 2 with only the perceptual session in which the participants were pre-informed of which hand each stimulus would be delivered to. In other words, we tested whether the perceptual kappa effect could be replicated even when participants could expect which hand would be stimulated. The mean result for each condition is shown in Fig. 3. Almost the same tendency as in Experiment 1, i.e., the perceptual kappa effect, was found. Indeed, a two-way repeated-measures ANOVA showed that the location, *F*(1.95, 29.24) = 16.919, *p* < 0.001, η_*p*_^2^ = 0.530, as well as the standard effect, *F*(1.56, 23.43) = 637.706, *p* < 0.001, η_*p*_^2^ = 0.977, was significant, whereas the interaction was not significant, *F*(3.5, 52.56) = 0.811, *p* = 0.510, η_*p*_^2^ = 0.051. The Scheffé contrasts revealed the same results as in Experiment 1. Moreover, a one-way repeated-measures ANOVA showed that the mean spatial-effect amount, presented in Fig. 4, did not significantly change with the lengthening of the standard, *F*(2, 30) = 0.560, *p* = 0.577, η_*p*_^2^ = 0.036.

## Discussion

The main purpose of the present study was to examine whether the spatiotemporal interactions as in the kappa effect would also occur in motor action. The results of the two experiments can be summarized as follows: (1) The reproduced duration was longer when two stimuli were delivered to different hands than when delivered to an identical hand, indicating the perceptual kappa effect, in the passive session of Experiment 1; (2) This increment of the reproduced duration remained at a constant amount regardless of the standard duration; (3) The reproduced duration was shorter when different hands were used than when an identical hand was used for two button pushes, except for the standard of 500 ms, in the active session of Experiment 1; (4) This decrement of the reproduced duration grew linearly with the standard duration; (5) The perceptual kappa effect occurred even when the participants were informed in advance regarding which hand would be stimulated in Experiment 2, and thus it did not seem plausible to attribute the differences between the results of the perceptual and those of the motor session in Experiment 1 to the participant’s expectation as well as the resultant attentional focus^19^,^20^,^21^ on the hand that would be stimulated/moved.

These results indicate the existence of spatiotemporal interactions in motor action, which might be evidence against the second hypothesis that we mentioned in the Introduction. Indeed, it predicated no differences between the with-space vs. without-space conditions in the motor session. However, the first hypothesis predicting that both perceptual and motor spatiotemporal interactions would cause the same spatial effects on time keeping was not completely supported by the current results because the effects of the standard duration differed between the perceptual and the motor session. The size of the perceptual kappa effect, expressed by the increment amount of the reproduced duration, is constant regardless of physical duration to be perceived. A given space thus stretches the perceived duration by an invariable amount. However, the decrement of the reproduced duration, resulting from a given space during motor action (reproduction), grows linearly with the physical duration to be reproduced. This suggests that a given space increases the “rate” of an internal timer^23^,^24^; because each tick of the timer is accelerated, the total error that leads to an earlier push of the second button is increased with the physical duration to be reproduced. Therefore, the two types (perceptual and motor) of spatiotemporal interactions might be based on independent mechanisms.

Nevertheless, the current results may be explained within a single theoretical framework, called the pacemaker-counter model, as posited by previous psychophysical studies^25^,^26^,^27^,^28^. In the pacemaker-counter device, the first module periodically emits a pulse, which is accumulated in the second module^24^. The number of accumulated pulses corresponds to the perceived duration. Non-temporal factors influence the process of switching on and off the device as well as the pace of emitting pulses^23^,^24^,^29^. In perception, a given space may delay the timing of switching off the device by a constant amount, and thus the incremental number of accumulated pulses is constant regardless of the physical duration to be perceived. In motor action (during reproduction), a given space may accelerate the pace of emitting pulses so that the total error is increased with the physical duration to be reproduced. The pacemaker-counter model thus helps to elucidate each type of spatiotemporal interaction. However, the fact that the same theory can apply does not necessarily indicate the same neural basis underlying both types of spatiotemporal interactions.

One of the factors that influence the process of switching on and off the pacemaker-counter device is attention^23^,^29^. Spatial attention might have been shifted from the first stimulus to the second one when these stimuli were delivered to different hands in the perceptual session, resulting in the delay in switching off the device. However, the perceptual kappa effect occurred even when the participants could expect where each stimulus would be presented in Experiment 2; this foreknowledge should have accelerated the shift in attention if it had worked as a prime in the Posner paradigm^30^,^31^. Moreover, it is difficult to posit that the spatial attention that was captured by the first stimulus could influence the detection of the second stimulus that was separated from the first one by 1000 ms or longer^31^,^32^. Nevertheless, it might be possible to attribute the delay in switching off the device to the loss of attentional resources, instead of the time required for the shift in attention. More attentional resources might be necessary for the binding of the second stimulus into the first one across time when the location of the second stimulus was different from that of the first one than when these locations were identical^33^,^34^. This situation then resulted in fewer resources for switching off the device.

The pace of emitting pulses is influenced by the arousal level^23^,^24^,^29^, but there is no reason to posit that the arousal level differed among the spatial conditions in the motor session. Although speculative, the pace of emitting pulses might be correlated with the activation level of some brain neurons^23^,^35^. The activation of brain areas such as the sensorimotor cortex, the parietal cortex, and the cerebellum is increased during bimanual movements compared with unimanual ones^36^,^37^,^38^,^39^,^40^. Incremental activation in these areas might accelerate the pace of emitting pulses when different hands were used for the two button pushes. Moreover, the parietal cortex might also be involved with the delay in switching off the device. Indeed, this area is related to a feature binding based on spatial attention^34^,^41^ as well as temporal attention^42^.

Most of the previous studies testing the perceptual kappa effect adopted three successive stimuli marking two neighboring intervals on each trial^5^,^6^,^7^,^11^ or adopted single intervals of a much longer duration (7 s or longer)^8^,^43^ than the present study. However, the perceptual session of Experiment 1 in the present study was designed on the basis of Grondin *et al.*^13^, who used only the standard duration of 500 ms. That previous study adopted the discrimination task, whereas the present study adopted the reproduction task. Despite this difference, both studies showed a similar size of the kappa effect. The mean spatial-effect amount was approximately 71 ms when the standard was 500 ms in the perceptual session of Experiment 1. The data of Grondin *et al.* indicated that the empty duration was perceived as being approximately 65 ms longer when two stimuli were delivered to different hands than when both were delivered to the same hand (in a condition where the two hands were placed near each other, as in the present study). The results of the present study also appeared consistent with what was reported by Grondin^21^ with sub-second single intervals in the visual modality (Experiment 2 in the article). These intervals were marked by two flashes, each of which was located on either the left or the right hemi-field. The data indicated that the empty duration was overestimated when the two flashes were located on different hemi-fields than when both were located on the same hemi-field (in a condition where the spatial condition was varied randomly across trials). Moreover, in an experiment by Price-Williams^8^ with the visual modality (Experiment I in the article), the incremental amount of the reproduced duration that resulted from the kappa effect was almost constant against the standard duration between 7 and 11 s, consistent with the results of the present study with a range between 500 and 1500 ms.

The results of the present study may be compared with a Bayesian model that was proposed by Goldreich^44^ to explain several spatiotemporal effects including the kappa effect in the tactile modality. This model posits the prior knowledge that two successive stimuli are perceived as a single object moving *slowly* between two locations. In other words, slow motion is expected for a succession of two stimuli. When the Bayesian observer (perceptual system) is confronted with a rapid succession of stimuli as in the kappa effect, this observer increases the perceived duration so that the single object is perceived as moving slowly. According to this prior, the kappa effect would be reduced when the physical duration to be perceived is lengthened while the spatial distance is kept constant. In this case, slower motion is physically (already) given, and there is no need to adjust the perceived duration. Indeed, a formula provided by the model (Equation 16 in Goldreich^44^) indicates that the kappa effect is reduced when the physical duration is lengthened, as shown in Fig. 5. In the present study, the lengthening of the standard duration magnified the decrement of the reproduced duration for the motor session, whereas it led to no significant effects for the perceptual session of Experiments 1 and 2. Nevertheless, the lengthening of the standard from 500 to 1500 ms might be too small to modulate the perceptual kappa effect significantly, given that a few participants (two for the passive session of Experiment 1 and four for Experiment 2) exhibited a negative value of the spatial-effect amount when the standard was 1500 ms, but no participants did when the standard was 500 ms. Future research should examine whether and how a further lengthening of the standard duration modulates each type of spatiotemporal interaction (although the increase in the standard from 7 to 11 s did not magnify the perceptual kappa effect in Price-Williams^8^, with the visual modality).

The perceptual kappa effect occurred whether or not the participants were informed in advance which hand would be stimulated (in the passive sessions of Experiments 1 and 2). This finding was inconsistent with the report of Grondin^21^. In his study, spatial effects disappeared when the first of the two flashes marking the empty duration was always delivered from an identical location so that participants could have foreknowledge of the first stimulus location. This inconsistency may be because Grondin’s study and the present study differed in the technical procedures used to inform the participants of the stimulus locations. The present study may rather be compared with Newman and Lee’s study using visual stimuli^43^. In their experiment, the kappa effect occurred when participants were informed of stimulus locations in advance by the experimenter; this finding is consistent with the results of Experiment 2 in the present study. However, they also found no kappa effect when participants were not informed of the stimulus locations, inconsistent with the results of Experiment 1. They used 9-s and 10-s intervals, which were much longer than those used in the present study. The effects of foreknowing spatial locations might interact with the duration range of empty intervals or with the sensory (tactile vs. visual) modality.

In summary, the perceptual and the motor spatiotemporal interactions lead to different behavioral phenomena in duration reproduction. In the perceptual case, a given space extends the perceived duration by an invariable amount, resulting in a constant increment of the reproduced duration regardless of the physical duration to be perceived. In the motor case, a given space causes the shortening of the reproduced duration if a supra-second duration is reproduced. More specifically, this decrement effect is increased with the physical duration to be reproduced, suggesting an increase in the rate of an internal timer. Whereas these results could be explained by positing a single pacemaker-counter mechanism, future research should examine the detailed psychophysical features of each type of spatiotemporal interaction. Moreover, neurophysiological techniques may be necessary to identify the source of each type of spatiotemporal interaction.

## Methods

### Participants

In total, 27 volunteers, aged 20–39 years, were recruited from Yamaguchi University. Sixteen (8 females) participated in Experiment 1. Sixteen (9 females), including 5 of those who also participated in Experiment 1, participated in Experiment 2. Consequently, the data from 16 participants were analyzed for each experiment. All participants self-reported being right handers. Written informed consent was obtained from each participant in accordance with procedures approved by the ethics review board of Yamaguchi University. Both experiments were conducted in accordance with the approved guidelines and regulations.

Two additional participants were recruited, but their data were not kept for the analysis. One, recruited for Experiment 1, exhibited a result (reproduced duration) of below 900 ms when the standard duration was 1500 ms for all location conditions in the passive session. The result was at least greater than 1100 ms for the other participants (see the Results section for the 95% confidence intervals between participants). The other, recruited for Experiment 2, frequently reported being unable to perceive the stimuli (thus, the session was restarted) and reported sleeping frequently during the experiment.

### Apparatus and stimuli

Empty time intervals were delimited by two tactile stimuli that were produced by solenoid vibrators (Uchida Denshi FD-2010-1). A cuboid contactor including a solenoid was attached to the back of each hand, being laid along the second metacarpal bone (Fig. 2). This stimulus location was adopted because the contactor was close to the index finger but did not disturb the movement of this finger for reproduction. Each of the two stimuli consisted of only one cycle of vibration with a period of 1 ms; thus, each stimulus lasted 1 ms. We measured that the peak amplitude of vibration in each solenoid was approximately 0.04 mm under the condition that the contactor was not in contact with any objects. At the beginning of the experiment, we verified that the participants reported perceiving each stimulus clearly. To mask the sounds produced by the solenoid vibration, the participants wore earplugs and headphones presenting a noise of 65 dB SPL during the task. The timing of activating the solenoids was controlled by a programmable pulse generator (AMPI Master 9).

Each participant was seated in front of a desk on which two buttons and a computer display were placed in an experimental booth. The index fingertip of the left hand and that of the right hand were placed on the left and the right button, respectively. These buttons were located 3.6 cm apart from each other. In Grondin *et al.*^13^, the perceptual kappa effect occurred regardless of whether the two hands were located near to or far from (3 foot) each other. In other words, an increasing separation between the two hands had no substantial effects on magnifying the kappa effect, whereas the occurrence of the effect only depended on whether the two stimuli were delivered to the same hand or to different hands. (see also Kuroda and Grondin^45^, who reported no effects of separation between the two fingers stimulated). The participant was instructed to always make the pad of the index finger contact the surface of the button. The participant was also instructed regarding the approximate location on which the thumb was to be placed and that on which the middle finger was to be placed (approximately 5 cm apart from the button), to keep the hand positions constant. Both hands were cupped so that the index finger could be moved easily and the hand posture maintained comfortably. A flat plate was placed above the hands to block visual information regarding the stimulus locations. Each button could be depressed by a power above 1.6 N. Pushing down the button produced voltage, which was recorded online by a data logger (Biopack MP100A). This record was monitored by an experimenter outside the booth. When the participant finished the reproduction, the experimenter operated the computer to advance to the next trial.

### Procedure

#### Experiment 1

Participants were instructed to reproduce the standard duration that was delimited by the onsets of two successive stimuli (i.e., the inter-onset interval). The standard duration was 500, 1000, or 1500 ms. Each trial began with the presentation of two letters on the computer display, indicating which hand had to be used for each button push. For example, “LR” instructed to use the left hand for the first button push (push down the left button first) and then the right hand for the second button push (push down the right button second). The stimulus presentation began 2–3 s after the letter presentation.

**Figure 1:**
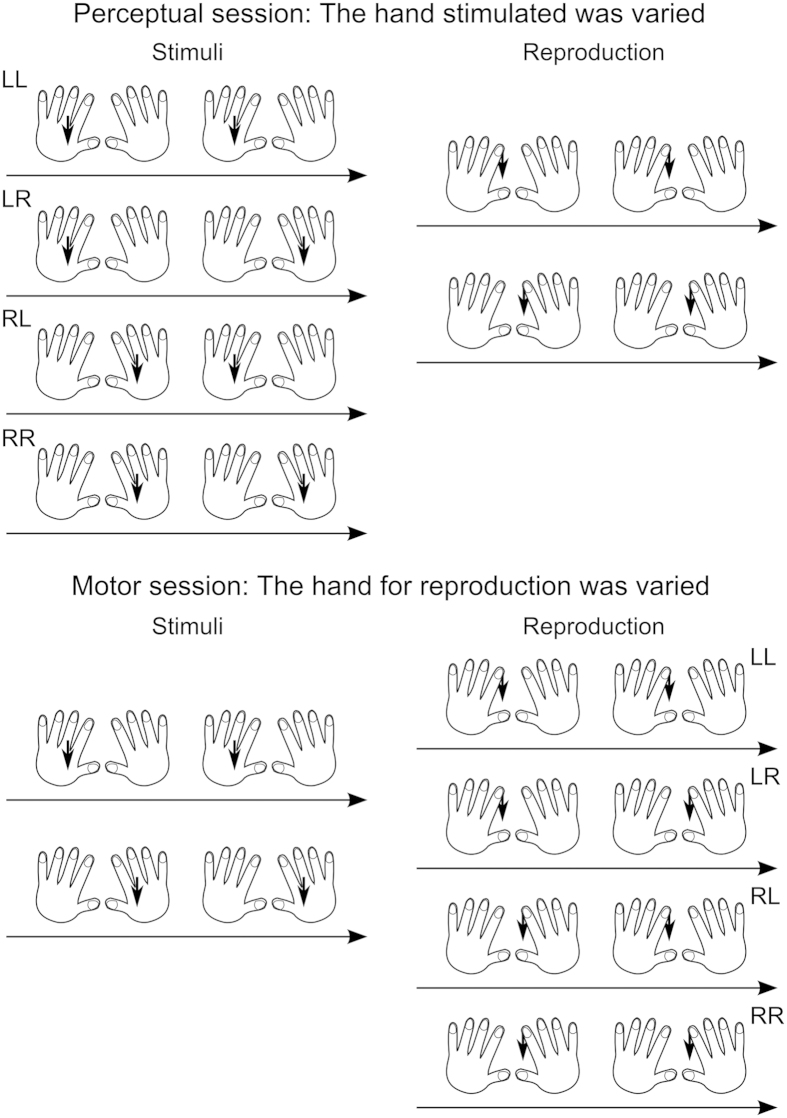
Schematic explanation of the perceptual session (upper) and the motor session (bottom) in Experiment 1. The hand to which each of the two stimuli was delivered was varied in the perceptual session, while the hand to be used for each of the two button pushes during the reproduction was varied in the motor session. The location of each stimulus and the hand (index finger) moved for the reproduction are marked with arrows. Each session was divided into two sub-sessions, according to which hand was used for both button pushes in the perceptual session and according to which hand both stimuli were delivered to in the motor session.

**Figure 2:**
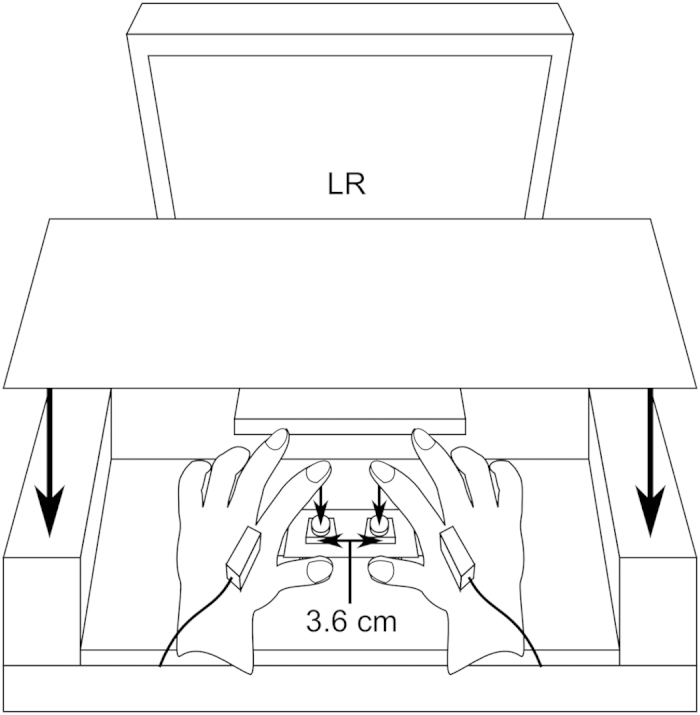
Experimental settings. Each stimulator was attached to the back of each hand, being laid along the second metacarpal bone. Two buttons were separated by 3.6 cm. The pad of the index finger made contact with the surface of the button. A flat plate was placed above the hands. Two letters on the display indicated the hand to be used for each button push for reproduction in Experiment 1 and indicated the hand that each stimulus would be delivered to in Experiment 2.

**Figure 3:**
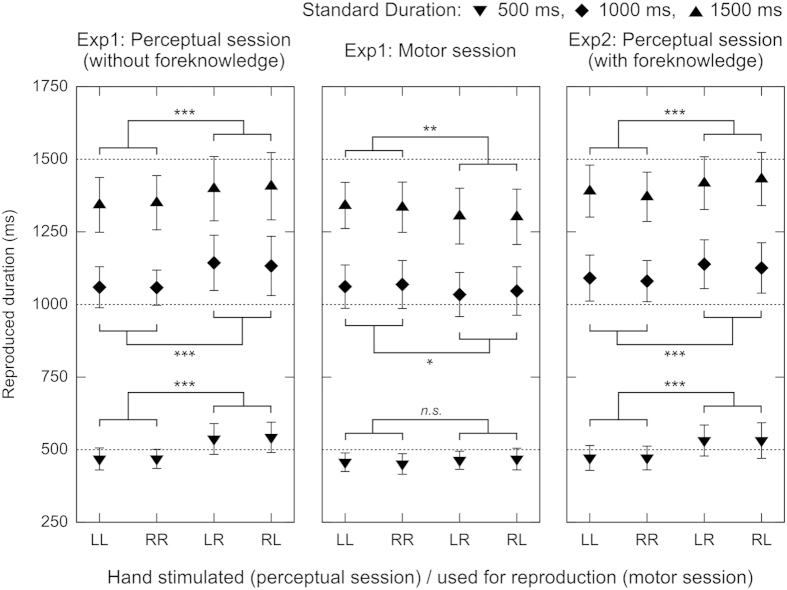
Mean reproduced duration for each experimental condition in the perceptual session of Experiment 1 (left), the motor session of Experiment 1 (middle), and Experiment 2 with only the perceptual session (right). Bars represent the 95% confidence intervals between participants. The results of the post hoc contrasts according to the Scheffé method testing the null hypothesis 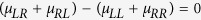 are also shown (*p < 0.05, **p < 0.01, ***p < 0.001). Because the interaction was not significant for the perceptual session of Experiment 1 and of Experiment 2, the three standards were pooled when the contrasts were conducted.

**Figure 4:**
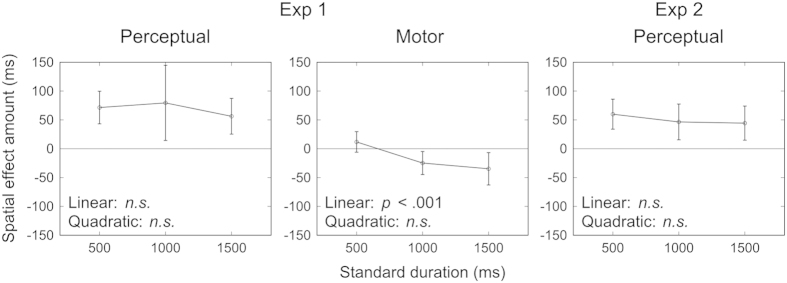
Mean spatial-effect amount, i.e., 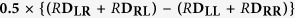, against the standard duration for the perceptual session of Experiment 1 (left), the motor session of Experiment 1 (middle), and Experiment 2 with only the perceptual session (right). Bars represent the 95% confidence intervals between participants. The results of the trend analysis for the linear and quadratic trends are also shown.

**Figure 5:**
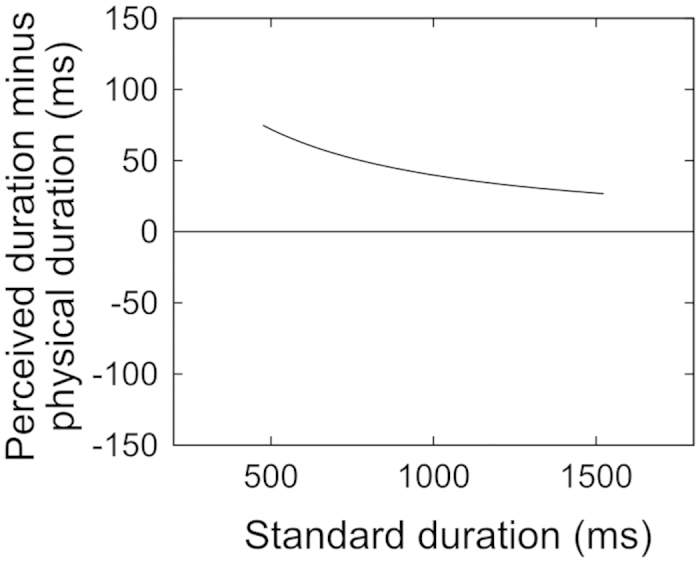
Duration overestimation resulting from the kappa effect against the standard duration, predicted from a Bayesian model^44^. *t* (physical duration) corresponding to *t’* (perceived duration) was estimated with Equation 16 in Goldreich^44^. In this equation, *σ*_*s*_ (spatial acuity) was fixed at 1 mm as in Goldreich. *σ*_*t*_ (temporal acuity) was increased in proportion to the standard with a Weber fraction of 16%^13^ according to the scholar property^26^. *σ*_*v*_ (degree of expectation of slow motion) was fixed at 4 cm/s, and this was determined arbitrarily so that the resulting values were close to the mean results found in the perceptual sessions of Experiments 1 and 2. Note that the exponential-like decrease that is apparent in this figure is still observed even if *σ*_*t*_ is fixed at a constant value.

The experiment consisted of two sessions (Fig. 1). In the perceptual session, each of the two stimuli was delivered to either the left or the right hand, resulting in four conditions, left-left, left-right, right-left, and right-right. An identical hand was used for reproduction (for both the first and second button pushes), and this perceptual session was divided into two sub-sessions according to whether the left or the right hand was used for both button pushes. In the motor session, each of the two button pushes was assigned to either the left or the right hand, resulting in four conditions, left-left, left-right, right-left, and right-right. Both stimuli were delivered to an identical hand, and this motor session was divided into two sub-sessions based on whether both stimuli were delivered to the left or the right hand. Thus, each session consisted of two sub-sessions. The order of the two sub-sessions, as well as that of the two sessions, was counterbalanced across participants. Each sub-session contained 5 blocks between which a break of a few seconds was taken. In each block, twelve experimental conditions (= 3 standards × 4 locations) were presented three times each in random order, yielding 36 trials. Because the first block of each sub-session was regarded as practice and not kept for the data analysis, twelve reproductions (=4 blocks × 3 trials) were obtained for each condition for each sub-session.

Participants were asked not to consider the spatial distance for reproduction, i.e., not to adjust their perceived duration or reproduction voluntarily based on the spatial distance between the hands that were stimulated or used for reproduction. They were also instructed not to count the cadence or make sounds synchronized with the stimuli (e.g., finger tapping and internal voicing).

#### Experiment 2

The same procedure as in Experiment 1 was adopted, but the experiment was restricted to the perceptual session. Moreover, the two letters presented at the beginning of the trial indicated the hand to which each of the two stimuli would be delivered (instead of indicating the hand to be used for reproduction as in Experiment 1). For example, “LR” indicated that the first stimulus and the second stimulus were delivered to the left and the right hand, respectively. Participants were instructed not to ignore these letters.

### Data analysis

As mentioned earlier, the first block of each sub-session was regarded as practice and not kept for the data analysis. For the following blocks, approximately 4% of the trials were removed from each session of Experiment 1 and 4% from Experiment 2 because (1) participants used incorrect hands; (2) they responded before the end of the stimuli; (3) the number of taps was incorrect; or (4) the reproduced duration fell into an outlier (a *far-out* case called by Tukey^46^) that was below 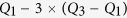 or above 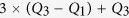. 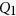 and 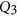 represent the first and the third quartile, respectively, estimated from the reproduced durations for each condition for each sub-session for each individual. The dependent variable was given by averaging the reproduced durations for each condition and by averaging the two sub-sessions for each session.

A repeated-measures ANOVA according to a 3 (duration) × 4 (location) design was conducted separately on the two sessions of Experiment 1. This ANOVA was also conducted for Experiment 2 (only the passive session). For each ANOVA, the *F* distribution was estimated with the degrees of freedom adjusted with the Greenhouse-Geisser epsilon when the sphericity assumption was not tenable (Mauchly’s test, *p* < 0.10).

The Scheffé method was adopted as the post hoc contrasts for the location effect. As mentioned in the Introduction, a longer reproduced duration was expected in the with-space (LR and RL) than the without-space (LL and RR) conditions when the kappa effect occurred, so that the null hypothesis positing 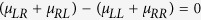 was tested. In addition, the null hypothesis positing 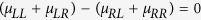 was tested; rejecting it indicates the effects of the first stimulus location for the perceptual session or of the first pushed button for the motor session. Furthermore, the null hypothesis positing 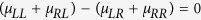 was tested; rejecting it indicates the effects of the second stimulus location or pushed button.

An additional analysis was conducted for each experiment. The amount of spatial effects was estimated using the equation 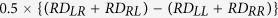 for each individual (*RD*, reproduced duration), to test how the amount of reproduction error resulting from a given space (LR and RL) relative to no space (LL and RR) was changed as a function of the standard duration. A one-way repeated-measures ANOVA for three levels (standards) was conducted on each session. The trend analysis was adopted to test a functional relationship between the spatial-effect amount and the standard duration.

## Additional Information

**How to cite this article**: Kuroda, T. and Miyazaki, M. Perceptual versus motor spatiotemporal interactions in duration reproduction across two hands. *Sci. Rep.* **6**, 23365; doi: 10.1038/srep23365 (2016).
